# Cytotoxic T-Cell-Based Vaccine against SARS-CoV-2: A Hybrid Immunoinformatic Approach

**DOI:** 10.3390/vaccines10020218

**Published:** 2022-01-30

**Authors:** Alexandru Tirziu, Virgil Paunescu

**Affiliations:** 1 Faculty of Medicine, “Victor Babes” University of Medicine and Pharmacy, 300041 Timisoara, Romania; vpaunescu@umft.ro; 2 Center for Gene and Cellular Therapies in the Treatment of Cancer Timisoara-OncoGen, Clinical Emergency County Hospital “Pius Brinzeu” Timisoara, No. 156 Liviu Rebreanu, 300723 Timisoara, Romania; 3 Immuno-Physiology and Biotechnologies Center, Department of Functional Sciences, “Victor Babes” University of Medicine and Pharmacy, No. 2 Eftimie Murgu Square, 300041 Timisoara, Romania

**Keywords:** SARS-CoV-2 T-Cell vaccine, epitopes, cytotoxic T lymphocytes, long term immunity, acquired immunity, molecular docking, in silico, protein folding, immunoinformatics, synthetic long peptide

## Abstract

This paper presents an alternative vaccination platform that provides long-term cellular immune protection mediated by cytotoxic T-cells. The immune response via cellular immunity creates superior resistance to viral mutations, which are currently the greatest threat to the global vaccination campaign. Furthermore, we also propose a safer, more facile, and physiologically appropriate immunization method using either intranasal or oral administration. The underlying technology is an adaptation of synthetic long peptides (SLPs) previously used in cancer immunotherapy. The overall quality of the SLP constructs was validated using in silico methods. SLPs comprising HLA class I and class II epitopes were designed to stimulate antigen cross-presentation and canonical class II presentation by dendritic cells. The desired effect is a cytotoxic T cell-mediated prompt and specific immune response against the virus-infected epithelia and a rapid and robust virus clearance. Epitopes isolated from COVID-19 convalescent patients were screened for HLA class I and class II binding (NetMHCpan and NetMHCIIpan) and highest HLA population coverage (IEDB Population Coverage). 15 class I and 4 class II epitopes were identified and used for this SLP design. The constructs were characterized based on their toxicity (ToxinPred), allergenicity (AllerCatPro), immunogenicity (VaxiJen 2.0), and physico-chemical parameters (ProtParam). Based on in silico predictions, out of 60 possible SLPs, 36 candidate structures presented a high probability to be immunogenic, non-allergenic, non-toxic, and stable. 3D peptide folding followed by 3D structure validation (PROCHECK) and molecular docking studies (HADDOCK 2.4) with Toll-like receptors 2 and 4 provided positive results, suggestive for favorable antigen presentation and immune stimulation.

## 1. Introduction

SARS-CoV-2 is an RNA virus responsible for the current COVID-19 pandemic. COVID-19 clinical features depend on the genetic variants of both the patient and the virus, inoculum size, and the presence of comorbidities [1]. The main transmission routes are respiratory and oral [2], suggesting the importance of mucosal immunity in disease onset. Even though several bioactive molecules undergoing clinical trials show positive results, they mainly work in the replicative phase of SARS-CoV-2 infection [3,4]. Hence, prevention via vaccination remains the cornerstone for ending the current viral pandemic.

Results from previous studies related to previous endemic outbreaks involving SARS-CoV and MERS-CoV suggested that coronaviruses trigger T cell and antibody immune responses in infected patients. However, antibody levels seem to become undetectable 2–3 years after recovery [5], while SARS-CoV-specific memory T-cells were identified after more than a decade post-infection. In 2016, Ng et al. isolated memory T cells 11 years after the infection [6]. In addition, le Bert et al. in 2020 discovered a viral-specific cellular immune response that lasted more than 17 years after SARS-CoV infection [1].

Analysis of the cellular and immune response from COVID-19 convalescent patients revealed mild COVID-19 subjects presented a vigorous T-cell mediated response months after SARS-CoV-2 infection and a low to undetectable titer of antibodies [7]. In contrast, severe COVID-19 presented an early phase lymphocytopenia followed by intense macrophage cytokine release [8] and high antibody titers due to poor viral clearance [9]. In the study performed by Sekine et al., 28% of healthy individuals presented cross-reactive memory T-cells against SARS-CoV-2 [7].

These findings confirm the bipolar role of T cells in COVID-19 pathogenesis—a higher number of lymphocytes during the initial phase of infection assures a rapid and efficient antiviral response, whereas an early lymphocytopenia followed by a subsequent immune hyperactivation leads to a poorer prognosis [8,10].

Lymphocytopenia is one of the most decisive factors in the evolution of COVID-19. Resolved lymphocytopenia predicts a favorable outcome, while unresolved low lymphocyte count leads to a poor prognosis. T lymphocytes were shown to hamper the innate immune response and prevent immune hyperactivation [9]. Thus, lymphocyte priming is a desirable event in the context of vaccination.

Mucosal immunity stimulation represents a promising alternative to traditional vaccination due to its simplicity, acceptance rate (less unpleasant than injections), and reduced postvaccinal complications. By targeting the pathogen right at the entry site, the viral replication decreases substantially, and the risk of evolution to more severe disease forms is mitigated [11,12]. At present, vaccination platforms range from live attenuated formulas to the newly introduced mRNA and viral vector-based platforms.

Peptide-based vaccination provides a promising alternative due to its high specificity, biological activity, tissue penetration, and low production costs [13,14]. Compared to single epitopes or protein subunits, SLPs (synthetic long peptides) present several advantages such as reduced CTL (cytotoxic T lymphocyte) tolerance, higher stability, T helper cell involvement, and enhanced peptide repertoire recognition.

Single class I-restricted epitopes diffuse systemically upon administration, bind randomly to CD8 molecules expressed on cytotoxic T cells, and consequently bypass the classical antigen-presenting pathway involving dendritic cells. As a result, a degree of CD8 anergy is achieved that might explain the disappointing results of single epitope vaccines [14,15]. In contrast, expanding the CD8^+^ T-cell epitope with a class II-restricted peptide sequence will force the resulting amino acid chain to be internalized and processed by the antigen-presenting cells. A proportion of peptides will be processed in the endosomal pathway and expressed with the HLA class II molecule, while others enter the vacuolar pathway to be cross-presented with the HLA class I molecule [15].

In terms of stability, single CD8^+^ T-cell epitopes bound to the MHC class I molecule are expressed for a short amount of time, leading to a weak and transient immune response. On the other hand, SLP-derived class I epitopes expressed higher stability when cross-presented. By including a class II-restricted epitope, T helper cell stimulation is produced with the subsequent cytokine release and immune response augmentation [14]. SLPs also have the advantage of combining peptide sequences located at distant sites inside the same protein or amino acid chains originating from different proteins. As a result, the immunogenic peptide repertoire is increased, providing a more specific immune response [15].

By expanding the peptide repertoire, SLPs can also function as immune response enhancers. Coppola et al. showed that administration of synthetic long peptides derived from Mycobacterium tuberculosis Latency Antigen Rv1733c, a protein expressed in dormant bacteria, led to an improved bacterial clearance in HLA-DR3 transgenic mice [16].

Due to their high data processing and analysis capacities, in silico methods can be a useful tool for characterizing diverse immunological events and speeding up the process of vaccine design. Immunoinformatic and computational biology approaches were already used for designing vaccines against infectious agents such as *Helicobacter pylori* [17], *Vibrio cholerae* [18], *Plasmodium* species [19], or *yellow fever virus* [20].

For COVID-19, research groups designed multi-epitope subunit protein vaccines [21,22], but this vaccine landscape lacks an SLP-based vaccine design.

Starting from the article published by Ng et al., who identified an anti-SARS-CoV cellular immune response even after 11 years post-infection [6] and based on our prior experience in personalized immunotherapy in cancer, on 15 January 2020, our group began working on a vaccine model that stimulated mainly the long-lasting T-cell-based immunity. As a result, the OncoGen research group finished its first in silico prediction of a T-cell-based vaccine adapted to Romanian phenotypic characteristics on 25 January 2020. The results were published on our website (https://oncogen.ro/ro/decode-project/) on 28 January 2020 and PrePrints on 6 February 2020 [23]. Following the previous study, the current technology uses a hybrid approach based on immunoinformatic methods described in the previous article, as well as data collected from databases containing epitopes from COVID-19 convalescent patients.

## 2. Materials and Methods

**Figure 1:**
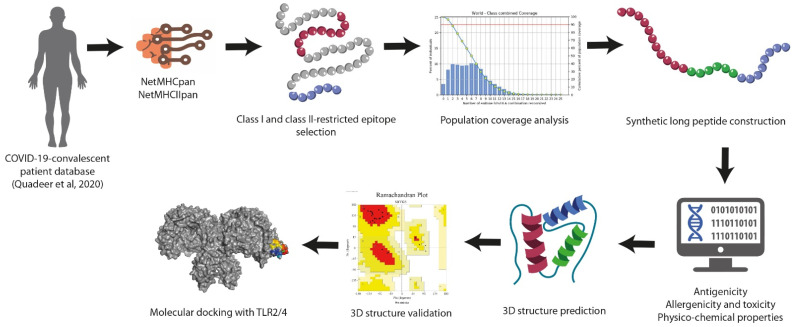
Schematic workflow followed for synthetic long peptide vaccine design.

The study workflow for designing SARS-CoV-2-specific synthetic long peptides can be visualized in Figure 1.

### 2.1. Epitope Screening

Class I and class II-restricted epitopes were extracted from a peptide pool comprising 1209 peptide sequences identified from 852 patients who recovered from COVID-19 [24]. The peptide pool database can be accessed here: https://www.mckayspcb.com/SARS2TcellEpitopes/ (accessed on 31 August 2021). Peptide screening was based on the following criteria:

- *Degree of conservation* (so that mutations identified in various SARS-CoV-2 will not influence the antigen processing and presentation significantly);
- *Cross-specificity*—multiple HLA allele coverage.

The most selective step in the antigen-presentation pathway is the interaction between peptides and HLA molecules. To screen the peptides with the highest binding potential for a certain HLA molecule, several machine-learning-based frameworks were used.

NetMHCpan and NetMHCIIpan are two artificial neural network-based methods trained on binding affinity and mass spectrometry peptidome data obtained from experimental results and deposited in IEDB (Immune Epitope Database) [25]. Both are available as online webservers and allow user input for epitope sequences, allele datasets, and HLA alleles in FASTA format. The output is provided as a percentile rank reflecting the likelihood that a given peptide will generate an immune response for a given HLA molecule. The percentile rank is obtained by comparing the results of the query epitope with results calculated for a pool of random peptides. In the case of NetMHCpan, strong binders are characterized by a percentile rank below 0.5%, while weak binders present a percentile rank below 2%. Alternatively, in NetMHCIIpan, strong binders have a percentile rank below 2%, while weak binders are considered below 10%.

### 2.2. Population Coverage Analysis

The peptide sets were screened using the IEDB population coverage tool, an algorithm that calculates the percentage of a population of interest that will be covered by a user-defined peptide-HLA dataset [26]. Further selection was performed so that the peptide pool would cover the maximum percentage of the population with the minimum number of class I and class II-restricted peptides.

### 2.3. Synthetic Long Peptide Construction

As previously described by Rabu et al., the synthetic long peptide construct with a higher probability to be presented and cross-presented to T cells by the dendritic cells comprises an HLA class II-restricted epitope at the N-terminus, a 6-mer cathepsin-sensitive linker sequence (LLSVGG), and an HLA class I-restricted epitope. The choice for this SLP construct was based on the following:

1. The HLA class II molecule is much more permissive in terms of epitope sequence length compared to the HLA class I molecule.
2. Class I-restricted epitope could undergo further cleavage by ERAP (endoplasmic reticulum aminopeptidase) in the presence of HLA class I molecule inside the endoplasmic reticulum, cleaving the remaining amino acids originating from the linker. As a result, peptides with 9-11 amino acids can fit perfectly to the HLA class I binding groove, as stated by the “molecular ruler” hypothesis [27].

The linker (LLSVGG) was designed by Rabu et al. to be cleaved by at least one of the main antigen-presenting cell endosomal cathepsins (L, D, and S). Experimental data performed on SLPs derived from tumor antigens showed a 100-fold increase in antigen presentation by using the LLSVGG linker compared to other linkers (GGGG, LVGS, LLSV, etc.) [15].

All possible combinations for the SLP constructs were generated based on class I and class-II restricted epitopes. All SLP candidates underwent a screening process based on predicted allergenicity, toxicity, physico-chemical properties, and immunogenicity.

### 2.4. Allergenicity Screening

Peptides may potentially elicit an IgE-mediated type I hypersensitivity reaction, especially in the case of mucosal contact. The allergic immune recognition of the peptide structures depends mainly on the amino acid sequence and three-dimensional structure. To rule out possible unpleasant reactions, allergenicity testing was performed using AllerCatPro, a web server that compares a query structure with FASTA sequences and 3D structures from an extensive allergen database of 4180 unique protein sequences. In this manner, both linear and discontinuous allergenic epitopes are detected. Similarity with gluten-derived allergens is analyzed using a gluten-like repeat pattern recognition algorithm [28].

### 2.5. Toxicity Screening

Toxicity was assessed using ToxinPred webserver (http://crdd.osdd.net/raghava/toxinpred/, accessed on 31 October 2021), a support vector machine algorithm that separates non-toxic peptides from the toxic peptides based on a training dataset containing peptides with less than 35 amino-acids extracted from various databases such as SwissProt and TrEMBL. The SVM-based algorithm depends upon amino acid or dipeptide composition of a given peptide, as well as motif identification [13].

### 2.6. Physico-Chemical Properties and Antigenicity

Peptide combinations with the most favorable physico-chemical properties were selected using the ProtParam library for BioPython. The instability index was calculated to assess the stability of a certain synthetic long peptide.

Based on the statistical analysis performed by Guruprasad et al., certain dipeptides occur more frequently in unstable proteins compared to stable ones [29]. By observing the stability of the 400 possible dipeptide combinations in the lab, the authors assigned for each molecule a weighted score. The instability index (II) is defined by the following formula:

$$II=\frac{10}{n}\overset{n-1}{\underset{i=1}{\sum }}DIWV\left(x_{i}x_{i+1}\right)$$

where *n*—number of amino acids in the sequence; *DIWV* (*x_i_x_i_*_+1_)—the instability weight value for the dipeptide starting in the position *i*; A value below 40 is considered stable, whereas an II above 40 is considered unstable.

VaxiJen webserver was used to identify which SLPs might elicit an immune response. VaxiJen is an alignment-independent immunogenicity prediction algorithm that uses auto-cross-covariance (ACC) transformation of protein sequences into vectors with equal lengths. The ACC algorithm is based on the principal component analysis (PCA) of the main 29 physico-chemical properties of amino acids represented by the z descriptors: z_1_ describing hydrophilicity, z_2_ molecular size, and z_3_ ionization status. The VaxiJen webserver (http://www.ddg-pharmfac.net/vaxijen/VaxiJen/VaxiJen.html, accessed on 30 November 2021) inputs a query sequence in FASTA format and outputs the probability that a certain protein is immunogenic. The main detected antigens can originate from bacteria, viruses, or tumors. For viral-derived antigens, the threshold score is 0.4—a value over 0.4 suggests probable antigenicity [30].

### 2.7. Three-Dimensional Structure Prediction

Rosetta ab initio was used to predict the three-dimensional conformations for each synthetic long peptide.

One of the main strategies for solving ab initio structures is using fragment libraries. A fragment library includes all favorable conformations that a specific 3-mer/9-mer can adopt, based on already solved structures uploaded on PDB (Protein Data Bank).

For each query peptide/protein, Rosetta ab initio approximates its secondary and tertiary structure by using libraries of 3-mer and 9-mer secondary structures. Generation of 3-mer and 9-mer libraries is performed using Robetta by searching the most probable 3-mer and 9-mer conformations in already solved structures.

Fragment library generation was performed using the Robetta webserver (http://old.robetta.org/fragmentsubmit.jsp, accessed on 30 November 2021). The user inputs the polypeptide FASTA sequence as a query for multiple sequence alignment algorithms with proteins from PDB. Fragment prediction is conducted using a hierarchical screening procedure that uses BLAST, PSI-BLAST, FFAS03, and 3D-Jury to detect homologous sequences, including distant evolutionary structures [31].

The 3-mer and 9-mer libraries are then used for ab initio protein folding by generating three-dimensional models consisting of fragments extracted from generated libraries so that physical interactions between the residues are favorable and the Rosetta score reaches its minimum value.

Rosetta score is calculated using the Rosetta scoring function, ref2015, which includes physics-based terms, such as electrostatic and van der Waals’ interactions, as well as statistical terms—the probabilities that a certain residue will adopt a specific conformation based on geometrical parameters [32].

Fragment assembly is done using knowledge-based potentials, which reflect the probability that backbone phi and psi angle values are conserved throughout evolution. The output is a low-resolution model that undergoes physics-based atomic refinement based on physical interactions between side chains [33].

### 2.8. Three-Dimensional Structure Validation

3D structure validation was performed to assess whether the 3D structure prediction provided stable, good-quality models. For this step, we used PROCHECK [34], and their corresponding Ramachandran plots were drawn. 3D structure visualization was performed using PyMol.

### 2.9. Molecular Docking Studies

To investigate how likely the SLPs are to be internalized by the antigen-presenting cells, molecular docking studies were performed using HADDOCK 2.4 [35,36]. The antiviral innate immune receptors TLR2 and TLR4 (Toll-like receptor) were used for this assay. By binding to Toll-like receptors 2 and 4, the subsequent cytokine release can trigger the internalization of the SLPs. Toll-like receptor three-dimensional structures were downloaded from the PDB database with the PDB ids 6NIG [37] and 3FXI [38], respectively.

The initial step for HADDOCK 2.4 molecular docking is that both the ligand and the receptor are treated as rigid objects in the tridimensional space. The algorithm searches for the most geometrically favorable surface for the ligand to bind to the receptor. The second step (it1) is a flexible docking protocol in which the torsion angles from the active residues of both the ligand and the receptor are modified to produce strong physical intermolecular bonds (hydrogen bonds, ionic interactions, etc.). The last step (itw) explores the capacity of the ligand to displace the water molecules surrounding the active site once bound to the receptor. This refining process adjusts the torsion angles so that the SASA (solvent-accessible surface area) is minimized. Scoring and ranking are performed during each docking stage, based on the HADDOCK score.

HADDOCK outputs several variables to describe the protein-ligand interaction: van der Waals energy, electrostatic energy, desolvation energy, restraints violation energy, and buried surface area. Based on these descriptors, the HADDOCK score is calculated as follows:

$$HADDOCK\;score=1.0E_{vdW}+0.2E_{elec}+1.0E_{desol}+0.1E_{AIR}$$

where: *E_vdW_*—van der Waals’ energy; *E_elec_*—electrostatic energy; *E_desol_*—desolvation energy; *E_AIR_*—restraints violation energy.

A negative HADDOCK score suggests a favorable interaction between the two docking partners.

In the it0 phase, 1000 models are generated, but only the top 200 models proceed to further docking steps. Each HADDOCK simulation generates 200 models for TLR2/4-SLP complex that are ranked, scored, and clustered based on structural similarity. The most reliable cluster has the lowest HADDOCK score and Z-score. The Z-score for a given model cluster indicates the number of standard deviations from the average cluster, suggesting that the best cluster has the most negative Z-score.

Further structure refinement was performed using HADDOCK 2.4 to reduce the RMSD, restraints violation energy, and to improve the HADDOCK score. Gibbs free energies and dissociation constants were calculated using the PRODIGY web server [39,40].

## 3. Results

### 3.1. 19 Peptides from Convalescent Patients Express High Degree of Conservation, Cross-Specificity and Bind Strongly to HLA Molecules

COVID-19 convalescent patient database contains 1209 peptide sequences and 843 distinct epitope-HLA pairs. Mean epitope conservation was 0.97. 87.2% of epitopes were recognized by a single HLA allele, and 12.8% by more than one allele.

Nineteen peptides were identified based on the degree of conservation >0.85, low percentile rank on NetMHCpan and NetMHCIIpan, and cross-specificity: 15 HLA class I-restricted and 4 class II-restricted. Most epitopes originated from the S protein (13/19, 68.42%) and M protein (4/19, 21.05%), probably due to their position on the viral surface membrane facilitating antigen recognition. (Table 1) Interestingly, peptides originating from the inner core, such as ORF1a or N were also identified in COVID-19-convalescent patients. By including them in the peptide pool, the expanded T-cell epitope repertoire can recognize viral targets presented by SARS-CoV-2-infected cells via the HLA class I pathway. Consequently, viral-infected cells will undergo lysis with subsequent viral clearance.

On artificial neural network testing with NetMHCpan and NetMHCIIpan, 81.5% of class I epitope-allele hits, and 85.7% class II epitope-allele hits are considered strong binders, whereas 18.5% of class I and 14.3% of class II epitope-allele pairs are weak binders. (Supplementary Tables S1 and S2) These findings support the use of this peptide set for further synthetic long peptide design.

**Figure 2:**
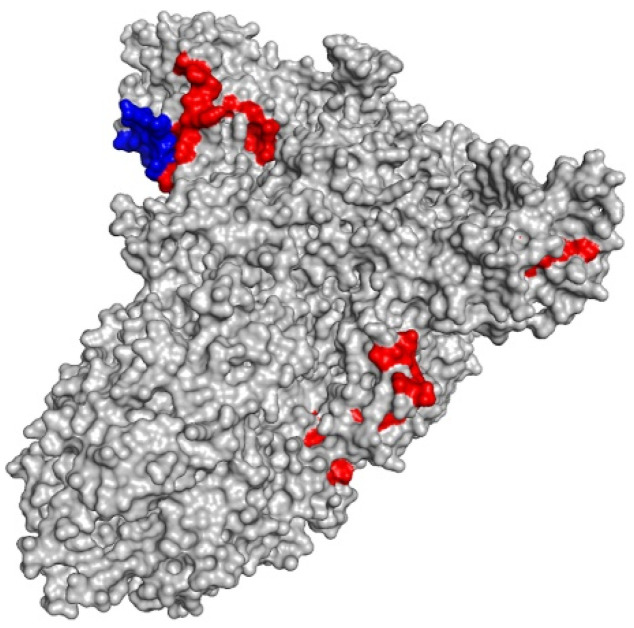
3D representation of the spike (S) protein (gray) with highlighted class I-restricted (red) and class II-restricted epitopes (blue).

Class I and class II-restricted epitopes can be visualized inside the spike (S) protein using PyMol (Figure 2).

### 3.2. 90% Probability That 2 Peptides Will Be Recognized by Any Individual

Population coverage analysis showed that the class I coverage was 85.94%, class II coverage 75.42%, and combined coverage 96.54%. The average number of epitope hits/HLA combinations recognized by the population was 4.49 for class I, 1.27 for class II, and 5.76 for the combined set. PC90 for the combined set was 1.81, which roughly translates to a probability of 90% that a minimum number of 2 peptides be recognized by any individual of the population (Table 2).

### 3.3. SLP Constructs Express High In Silico Immunogenicity and Are Stable under Laboratory Conditions

The SLP construct comprises an HLA class II and an HLA class I-restricted epitope joined by a cathepsin-sensitive linker (LLSVGG). The choice for this linker was made based on the experimental data of Rabu et al. on in vitro and in vivo antigen presentation assays.

Out of the 60 (15 × 4) possible combinations of synthetic long peptides, only 36 presented an instability index below 40 and a VaxiJen score above 0.4 (the threshold for viral antigens). VaxiJen score mean was 0.51, and the standard deviation was 0.07. The minimum value was 0.406, suggesting that all 36 constructs are, in theory, immunogenic. The mean instability index was 24.14, standard deviation 6.3, and the maximum value 35.92. An instability index below 40 suggests that the selected synthetic long peptides are highly likely to be stable under laboratory conditions (Supplementary Table S3).

### 3.4. Peptide Constructs Did Not Express Allergenicity nor Toxicity

One of the major problems in peptide-based therapeutics is the potential risk for toxicity or allergenicity. To exclude such inconveniences, we performed in silico toxicity analysis using ToxinPred webserver and allergenicity analysis using AllerCatPro webserver. Toxicity screening output revealed that all 36 constructs had a negative SVM score (mean = −1.3, standard deviation = 0.18), suggesting that the probability for SLPs to be toxic is unlikely. On allergenicity prediction, none of the SLPs expressed significant similarity with known allergens (Supplementary Table S4).

### 3.5. SLP Three-Dimensional Structure Prediction and Validation

For each synthetic long peptide structure, we predicted 200 three-dimensional models, which were clustered based on structural similarity and ranked based on the Rosetta score. The model with the best score was selected for further analysis (Supplementary Table S5).

**Figure 3:**
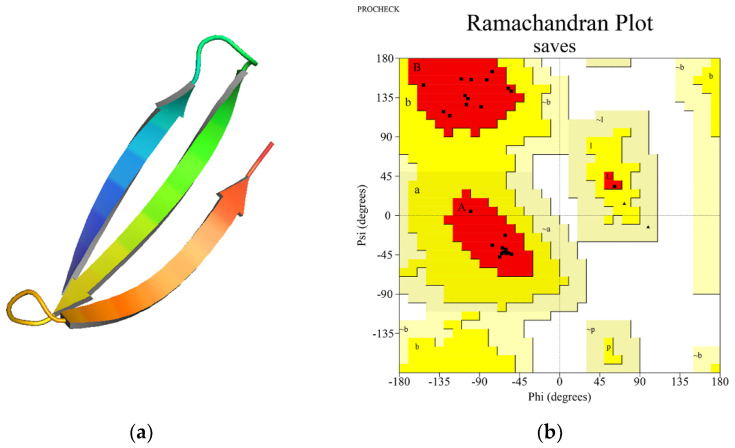
(**a**) 3D structure visualization of one synthetic long peptide, PINLVRDLPQGFSALLLSVGGWTAGAAAYY, using PyMol; (**b**) Ramachandran plot for the corresponding SLP. The dots representing the amino acids are mainly located in the most favorable regions (red) or the additional allowed regions (yellow). Most amino acids are located in the beta-sheet and alpha-helix regions.

Analysis of the Ramachandran plots revealed that the percentage of residues in the most favorable regions is above 90% for each three-dimensional peptide structure, suggesting good quality model predictions. The mean percentage of residues in the most favorable regions was 96.4%, while for the residues located in the additional allowed regions was 3.6%. None of the residues adopted unfavorable conformations. All Rosetta scores were negative, suggestive for theoretical thermodynamical stability. (Figure 3b and Supplementary Table S5) Structure visualization was performed with PyMol (Figure 3a).

### 3.6. Syntethic Long Peptides Present Favourable Interaction with Toll-Like Receptors 2 and 4

After we have identified that the selected peptides can elicit a cytotoxic T lymphocyte-mediated immune response, we wanted to investigate the capacity of synthetic long peptide constructs to initiate an innate immune response mediated by Toll-like receptors. Toll-like receptors 2 and 4 are membrane-bound proteins that recognize motifs belonging to viral structural and non-structural proteins. Therefore, by activating Toll-like receptors, synthetic long peptides might elicit an antiviral cytokine release aiding in viral clearance and antigen presentation.

To investigate the interaction between candidate SLPs and TLR2/4, molecular docking studies were performed using HADDOCK 2.4. For each SLP-TLR2/4 interaction, 200 models were generated after the itw stage. These models were grouped into clusters based on their structural similarity (RMSD < 2Å). The best cluster was chosen based on the most negative Z and HADDOCK scores. From the best cluster, the top model was chosen for further structure refinement (Supplementary Tables S6 and S7) and free energy calculation.

**Figure 4:**
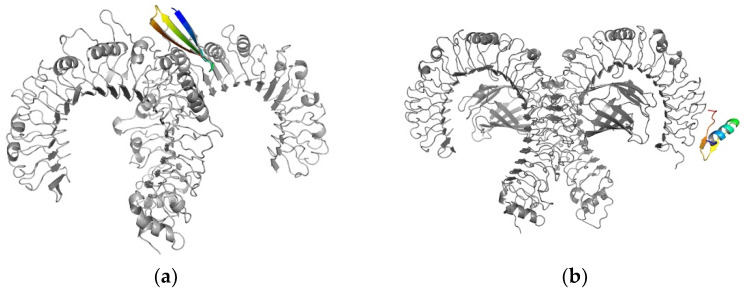
(**a**) One synthetic long peptide (colored)-toll-like receptor 2 (gray) docked complex with a binding energy of −12.5 kcal/mol (**b**) Toll-like receptor 4 (gray) in complex with a synthetic long peptide (colored) presenting a binding energy of −10.8 kcal/mol.

3D structure visualization was performed using PyMol (Figure 4).

The mean HADDOCK score was −107.41 for TL2/SLP and −110.064 for TLR4/SLP, while the minimum value was −141.3 for TLR2/SLP and −144.2 for TLR4/SLP. These findings suggest a favorable interaction between the two docking partners with the potential to trigger an innate immune response. In all SLP models, the main interactions responsible for Toll-like receptor binding are electrostatic. Statistical analysis has shown that electrostatic energies contribution is significantly higher than the van der Waals interactions (*p* < 0.001). When comparing the differences in van der Waals energies between SLPs and the two TLRs, we observed no significant difference between the two groups (*p* = 0.247). However, we found significant differences in the electrostatic energies between the two docking groups (*p* < 0.001). In the case of TLR2 docking, the desolvation change in energy had a small contribution to the overall HADDOCK score and was mainly negative, implying that water dissociates freely from the binding sites, allowing ligand-receptor binding. Compared to TLR2, TLR4 desolvation changes in energy were more negative, thus allowing water to rapidly dissociate from the docking interfaces and allowing ligand-receptor interaction. The mean restraints violation energy is 0.67 (TLR2) and 1.3 (TLR4), suggestive for good quality docking simulations.

ΔG and Kd values calculated using PRODIGY webserver predict a favorable interaction between the SLP set and the Toll-like receptors 2 and 4. The mean ΔG value for TLR2 was −11.475 kcal/mol, while for TL4 was −10.317 kcal/mol. The interaction between the SLPs and TLR2 was stronger than TLR4 interaction in terms of Gibbs’ free energy (*p* < 0.001) and dissociation constants (*p* < 0.001) (Supplementary Table S8). These findings could suggest that besides the specific immunity, SARS-CoV-2-specific synthetic long peptides also stimulate innate immunity. Theoretically, cytokine release triggered by TLR-binding would aid in antigen presentation and T-cell activation.

## 4. Discussion

Despite the growing number of vaccine technologies, the COVID-19 pandemic is not over yet. By using the cancer-derived SLP technology, a new vaccine platform might be implemented against infectious diseases, including COVID-19. SLPs function as a robust immune response trigger, but they can also enhance the efficacy of the already available vaccines by providing additional T cell epitope sets. Based on experimental observations by Rabu et al., the present study proposes an in silico model that might be used in the near future as a potential vaccination strategy for emerging infections.

This report shows how a synthetic long peptide-based vaccine can be produced using in silico tools. Compared to other studies, the presented workflow exploits a hybrid approach by processing data collected from COVID-19 convalescent patients.

A nineteen (15 class I and 4 class II) peptide pool was constructed using the data from a meta-analysis involving 852 COVID-19 patients worldwide, based on the degree of conservation and cross-specificity. Peptides were tested for HLA binding using the artificial neural networks NetMHCPan and NetMHCIIPan, where 81.5% (class I) and 85.7% (class II) of allele-epitope hits were identified as strong binders. Population coverage analysis identified a 90% probability that any individual who carries an HLA allele contained in the IEDB database can recognize at least 2 peptides. Class I coverage was 85.94%, while class II coverage was 75.42%, and combined coverage was 96.54%. Not all populations are equally represented, leading to data being biased to certain favored populations. To overcome this problem, further clinical studies are required to fully characterize the worldwide HLA haplotype occurrence and the recognized peptide repertoire.

Designed synthetic long peptides comprised a class II-restricted epitope, a cathepsin-sensitive linker, and a class I-restricted epitope. This design assures a bidirectional stimulation of both cellular and humoral immune responses with higher viral clearance.

36 out of 60 possible synthetic long peptide constructs expressed an instability index below 40 and a VaxiJen score below the viral threshold (0.4). These findings suggest that, in theory, the 36 SLP pool contains stable molecules with immunogenic potential.

Due to their low molecular weight, size, and degradation of vaccine components in the mucosal environment, peptides present lower immunogenicity compared to the traditional vaccine formulae. Therefore, adjuvants are needed as immune response enhancers to increase delivery to antigen-presenting cells. Subsequently, antibody titers, cytokine, and co-stimulatory molecule expression increase while the antigen dose decreases [41]. Vaccine components, when administered intranasally or orally with TLR agonists, such as poly(I:C), CpG-ODN, or PS-cGAMP exerted mucosal as well as systemic antigen-specific immune responses [12].

One of the major problems in peptide-based platforms involves potential allergenicity and toxicity. In silico studies provide a rapid, cost-effective method to screen for molecules with high allergenic or toxic potential. When performing toxicity analysis with ToxinPred, all 36 SLP constructs presented a negative SVM score, making them unlikely to exert any toxic effect. Allergenicity analysis using AllerCatPro revealed that none of the peptide constructs would elicit allergic reactions. However, in vitro and in vivo validation studies are necessary to screen for such potential unwanted effects [13,28,42].

Three-dimensional structure prediction provided good quality models, supported by the percentage of residues located in the thermodynamically most favorable regions.

Toll-like receptors are pattern-recognition receptors (PRRs) involved in innate immune system antigen recognition. TLRs are horseshoe-shaped transmembrane proteins with various locations such as the plasmalemma (recognizing extracellular pathogens) or the cytoplasm attached to vesicles (recognizing intracellular microorganisms). TLR dimerization assures a higher recognition repertoire comprising fungal, bacterial, or viral proteins. Recent studies demonstrated the involvement of TLR1, 2, and 6 in COVID-19-related cytokine storms by targeting the envelope protein. [43] Additionally, TLR4 was shown to be involved in an anti-bacterial-like early immune response by interacting with the SARS-CoV-2 spike protein [44].

Molecular docking studies performed with HADDOCK 2.4 identified high-affinity interactions between TLR2/4 and the predicted synthetic long peptide pool, which suggest a high likelihood for the SLPs to trigger an immune response with subsequent internalization and antigen processing inside the dendritic cells. When comparing the electrostatic with the van der Waals energies, we found that electrostatic energies contribute the most to the SLP-TLR2/4 interaction. There were no significant differences between the van der Waals energies of the TLR2-SLP group and the TLR4 one, but in the TLR2 group, the electrostatic energies were stronger and consequently improved the HADDOCK score, ΔG and dissociation constants. High similarities between the Gibbs free energies and dissociation constants inside the TLR2/4-SLP groups result in an even distribution of the SLPs at the administration site and quasi-equal binding probability to TLRs. Even though the presented in silico studies provide positive results, experimental validation is required to fully characterize the synthetic long peptide set in terms of immunogenicity, allergenicity, and toxicity. The 36 synthetic long peptide pool presents a considerable degree of redundancy which might be useful when performing experimental validation so that the initial class I and class II-restricted epitopes will not be lost.

This vaccine design relies on intranasal administration via droplets. This formula is preferred because of its simplicity (can be administered by any individual, therefore reducing the number of medical professionals required for vaccination), tolerability (compared to traditional vaccination, which some individuals find unpleasant or painful), and reduced number of complications. Additionally, intranasal vaccination provides mucosal immunity that targets viral particles right at the entry site [45,46].

SARS-CoV-2 infection is associated with lymphocytopenia and an inversely proportional innate immune cell count and cytokine concentrations with potentially life-threatening effects [43]. Therefore, immune priming with subsequent lymphocyte stimulation may be beneficial for preventing the evolution to severe COVID-19 [47]. In addition, it was shown that active T cells mitigate the overly active innate immune response in mice, providing an additional benefit for T cell stimulation [44].

In this in silico study, we presented an alternative vaccine design firstly described and tested on cancer immunotherapy. HLA class I and class II-restricted epitopes originating from COVID-19-convalescent patients were used for constructing synthetic long peptides that underwent further computational analysis. Based on our promising results on in silico models, in vitro, followed by in vivo studies are needed to validate our findings and investigate the immunogenic potential of our proposed design.

## Figures and Tables

**Table 1:** Selected HLA class I and class II-restricted epitopes based on their conservation and the number of allele hits.

Peptide Sequence	HLA Class	Start	End	HLA Alleles	Protein	Conservation
WTAGAAAYY	I	258	266	A *01:01, A *26:01, A *29:02, B *35:01	S	0.948138
LTDEMIAQY	I	865	873	A *01:01, A *29:02, B *35:01, C *07:02	S	0.99844
ATSRTLSYY	I	171	179	A *11:01, A *01:01, B *57:01	M	0.998295
LPPAYTNSF	I	24	32	B *53:01, B *35:01, B *07:02	S	0.969828
LSYFIASFR	I	93	101	A *11:01, A *31:01, A *68:01	M	0.998614
NSFTRGVYY	I	30	38	A *68:01, A *26:01, A *29:02	S	0.995425
TSNQVAVLY	I	604	612	B *57:01, A *26:01, B *35:01	S	0.999212
KTFPPTEPK	I	361	369	A *11:01, A *03:01, A *68:01	N	0.973513
VASQSIIAY	I	687	695	B *35:01, B *15:01, A *29:02	S	0.993504
CVADYSVLY	I	361	369	A *29:02, B *15:01, A *26:01	S	0.994539
GVYFASTEK	I	89	97	A *68:01, A *11:01, A *03:01	S	0.957055
RLFRKSNLK	I	454	462	A *31:01, A *03:01, A *11:01	S	0.995434
TISLAGSYK	I	1504	1512	A *68:01, A *11:01, A *03:01	ORF1a	0.987915
LPFNDGVYF	I	84	92	B *35:01, B *51:01, B *07:02	S	0.98813
AEIRASANL	I	1016	1024	B *40:01, B *44:02, B *44:03	S	0.99695
PINLVRDLPQGFSAL	II	209	223	DRB1 *03:01, DRB3 *01:01	S	0.878983
SRTLSYYKLGASQRV	II	173	187	DRB5 *01:01, DRB5 *01:02	M	0.997732
SYYKLGASQRVAGDS ITRFQTLLALHRSYL	II	177	191	DQA1 *05:01, DQB1 *03:01 DRB1 *01:01, DRB1 *07:01	M	0.998787
	II	235	249	DRB1 *01:01	S	0.983345

**Table 2:** Population coverage analysis for the combined peptide set.

	Coverage	Average Hit	PC90
Class I	85.94%	4.49	0.71
Class II	75.42%	1.27	0.41
Combined	96.54%	5.76	1.81

## Supplementary Materials

The following supporting information can be downloaded at: https://www.mdpi.com/article/10.3390/vaccines10020218/s1, Table S1. Predictions for class I-restricted epitopes using NetMHCpan. For each peptide-HLA allele combination, the EL (eluting ligand) and BA (binding affinity) percentile rankings are given. A percentile ranking below 0.5% is considered a strong binder, while a percentile ranking below 2% is considered a weak binder. Table S2. Predictions for class II-restricted epitopes using NetMHCIIpan. For each peptide-HLA allele combination, the percentile rankings are given. A percentile ranking below 2% is considered a strong binder, while a percentile ranking below 10% is considered a weak binder. Table S3. Candidate synthetic long peptides and their corresponding VaxiJen score, instability index (II) and molecular weight (Mol wt). Table S4. Predicted synthetic long peptide (SLPs) sequences, toxicity and allergenicity prediction. Toxicity prediction was performed using ToxinPred webserver, while allergenicity was predicted using AllerCatPro. Table S5. Synthetic long peptide sequences and their best Rosetta score. MFR—most favourable regions; AAR—additional allowed regions. Table S6. HADDOCK results for TLR2-SLP molecular docking (after refinement). Table S7. HADDOCK results for TLR4-SLP molecular docking (after refinement). Table S8. Predicted ΔG and Kd for the TLR2/4-SLP interactions using PRODIGY.
