# Supplementary material for: Cytotoxic T-Cell-Based Vaccine against SARS-CoV-2: A Hybrid Immunoinformatic Approach
Source: Vaccines (Basel). 2022 Jan 30;10(2):218. doi: 10.3390/vaccines10020218 (PMC8878688; doi:10.3390/vaccines10020218)
Supplement: Supplementary file 1 [file vaccines-10-00218-s001.zip › vaccines-1566136-supplementary.pdf]

Supplementary data for the article "Cytotoxic T-cell-based vaccine against SARS-CoV2: a hybrid immunoinformatic approach" by Alexandru Tirziu and Virgil Paunescu.

Table S1. Predictions for class I-restricted epitopes using NetMHCpan. For each peptide-HLA allele combination, the EL (eluting ligand) and BA (binding affinity) percentile rankings are given. A percentile ranking below 0.5% is considered a strong binder, while a percentile ranking below 2% is considered a weak binder.

Number	Peptide								
		HLA-A01:01		HLA-A26:01		HLA-A29:02		HLA-B35:01	
1	WTAGAAAYY	EL_Rank	BA_Rank	EL_Rank	BA_Rank	EL_Rank	BA_Rank	EL_Rank	BA_Rank
		0.0913	0.0166	0.0246	0.0075	0.1201	0.0261	0.6035	0.1907
		HLA-A01:01		HLA-A29:02		HLA-B35:01		HLA-C07:02	
2	LTDEMIAQY	EL_Rank	BA_Rank	EL_Rank	BA_Rank	EL_Rank	BA_Rank	EL_Rank	BA_Rank
		0.0014	0.0066	0.1849	0.5639	0.3419	0.5675	1.2973	3.0282
		HLA-A01:01		HLA-A11:01		HLA-B57:01			
3	ATSRTLSYY	EL_Rank	BA_Rank	EL_Rank	BA_Rank	EL_Rank	BA_Rank		
		0.0521	0.041	0.2399	0.2605	0.6159	0.9269		
		HLA-B07:02		HLA-B35:01		HLA-B53:01			
4	LPPAYTNSF	EL_Rank	BA_Rank	EL_Rank	BA_Rank	EL_Rank	BA_Rank		
		0.4334	0.6093	0.0912	0.1476	0.1225	0.3326		
		HLA-A11:01		HLA-A31:01		HLA-A68:01			
5	LSYFIASFR	EL_Rank	BA_Rank	EL_Rank	BA_Rank	EL_Rank	BA_Rank		
		0.6634	0.1129	0.2249	0.0186	0.2393	0.009		
		HLA-A26:01		HLA-B35:01		HLA-B57:01			
6	TSNQVAVLY	EL_Rank	BA_Rank	EL_Rank	BA_Rank	EL_Rank	BA_Rank		
		0.2075	0.8458	0.2621	0.3837	0.5504	1.5585		
		HLA-A03:01		HLA-A11:01		HLA-A68:01			
7	KTFPPTEPK	EL_Rank	BA_Rank	EL_Rank	BA_Rank	EL_Rank	BA_Rank		
		0.004	0.0548	0.0023	0.0212	0.2422	0.8989		
		HLA-A29:02		HLA-B15:01		HLA-B35:01			
8	VASQSIIAY	EL_Rank	BA_Rank	EL_Rank	BA_Rank	EL_Rank	BA_Rank		
		0.2279	0.759	0.1156	0.592	0.018	0.0263		
		HLA-A03:01		HLA-A11:01		HLA-A68:01			
9	GVYFASTEK	EL_Rank	BA_Rank	EL_Rank	BA_Rank	EL_Rank	BA_Rank		
		0.0337	0.0539	0.0242	0.0265	0.793	0.9521		
		HLA-A03:01		HLA-A11:01		HLA-A31:01			
10	RLFRKSNLK	EL_Rank	BA_Rank	EL_Rank	BA_Rank	EL_Rank	BA_Rank		
		0.006	0.0078	0.1843	0.1475	0.34	0.2274		
		HLA-A03:01		HLA-A11:01		HLA-A68:01			
11	TISLAGSYK	EL_Rank	BA_Rank	EL_Rank	BA_Rank	EL_Rank	BA_Rank		
		0.3619	0.1972	0.1763	0.0678	0.534	0.204		
		HLA-B07:02		HLA-B35:01		HLA-B51:01			
12	LPFNDGVYF	EL_Rank	BA_Rank	EL_Rank	BA_Rank	EL_Rank	BA_Rank		
		0.6256	1.1603	0.0145	0.0108	0.199	0.1041		
		HLA-B40:01		HLA-B44:02		HLA-B44:03			
13	AEIRASANL	EL_Rank	BA_Rank	EL_Rank	BA_Rank	EL_Rank	BA_Rank		
		0.0272	0.0147	0.1568	0.1003	0.1479	0.1147		
14	CVADYSVLY	HLA-A03:01		HLA-A11:01		HLA-A68:01			

		EL_Rank	BA_Rank	EL_Rank	BA_Rank	EL_Rank	BA_Rank
		0.0321	0.0164	0.0287	0.0146	0.5368	0.5148
15	NSFTRGVYY	HLA-A26:01		HLA-A68:01			
		EL_Rank	BA_Rank	EL_Rank	BA_Rank		
		0.1743	0.3832	0.9916	1.2382		

Table S2. Predictions for class II-restricted epitopes using NetMHCIIpan. For each peptide-HLA allele combination, the percentile rankings are given. A percentile ranking below 2% is considered a strong binder, while a percentile ranking below 10% is considered a weak binder.

Number	Peptide						
1	SYYKLGASQRVAGDS	HLA-DQA1*05:01-DQB1*03:01		HLA-DRB1*01:01		HLA-DRB1*07:01	
		Rank	Rank_BA	Rank	Rank_BA	Rank	Rank_BA
		1.74	2.43	1.22	0.21	1.76	1.64
2	PINLVRDLPQGFSAL	HLA-DRB1*03:01					
		Rank	Rank_BA				
		1.45	0.07				
3	SRTLSYYKLGASQRV	HLA-DRB5*01:01		HLA-DRB5*01:02			
		Rank	Rank_BA	Rank	Rank_BA		
		0.57	0.22	0.63	0.23		
4	ITRFQTLLALHRSYL	HLA-DRB1*01:01					
		Rank	Rank_BA				
		6.24	0.87				

Table S3. Candidate synthetic long peptides and their corresponding VaxiJen score, instability index (II) and molecular weight (Mol wt).

Sequence	VaxiJen score	Conclusion	II	Mol wt (g/mol)
PINLVRDLPQGFSALLLSVGGAEIRASANL	0.69	Probable ANTIGEN	32.57	3092.547
PINLVRDLPQGFSALLLSVGGATSRTLSYY	0.6754	Probable ANTIGEN	25.833333	3209.648
PINLVRDLPQGFSALLLSVGGTISLAGSYK	0.6082	Probable ANTIGEN	14.386667	3087.567
PINLVRDLPQGFSALLLSVGGKTFPPTEPK	0.6064	Probable ANTIGEN	33.3	3192.704
PINLVRDLPQGFSALLLSVGGTSNQVAVLY	0.5993	Probable ANTIGEN	15.933333	3142.602
ITRFQTLLALHRSYLLLSVGGATSRTLSYY	0.5852	Probable ANTIGEN	30.263333	3401.909
ITRFQTLLALHRSYLLLSVGGAEIRASAN	0.5733	Probable ANTIGEN	16.9	3171.65
SRTLSYYKLGASQRVLLSVGGTISLAGSYK	0.5715	Probable ANTIGEN	21.276667	3176.62
SRTLSYYKLGASQRVLLSVGGKTFPPTEPK	0.5698	Probable ANTIGEN	29.946667	3281.758
SRTLSYYKLGASQRVLLSVGGTSNQVAVLY	0.5626	Probable ANTIGEN	17.74	3231.656
PINLVRDLPQGFSALLLSVGGVASQSIIAY	0.5599	Probable ANTIGEN	34.116667	3099.577
SRTLSYYKLGASQRVLLSVGGLPFNDGVYF	0.541	Probable ANTIGEN	23.79	3308.738
PINLVRDLPQGFSALLLSVGGGVYFASTEK	0.5304	Probable ANTIGEN	19.413333	3149.593
SRTLSYYKLGASQRVLLSVGGVASQSIIAY	0.5232	Probable ANTIGEN	35.21	3188.631
SYYKLGASQRVAGDSLLSVGGAEIRASANL	0.521	Probable ANTIGEN	22.63	3054.37
ITRFQTLLALHRSYLLLSVGGTISLAGSYK	0.518	Probable ANTIGEN	18.183333	3279.828
ITRFQTLLALHRSYLLLSVGGKTFPPTEPK	0.5163	Probable ANTIGEN	22.243333	3384.965
ITRFQTLLALHRSYLLLSVGGTSNQVAVLY	0.5091	Probable ANTIGEN	19.73	3334.863
SYYKLGASQRVAGDSLLSVGGATSRTLSYY	0.5064	Probable ANTIGEN	19.73	3171.471
PINLVRDLPQGFSALLLSVGGLPPAYTNSF	0.496	Probable ANTIGEN	29.946667	3157.615
SRTLSYYKLGASQRVLLSVGGRLFRKSNLK	0.4954	Probable ANTIGEN	35.923333	3398.957

SRTLSYYKLGASQRVLLSVGGGVYFASTEK	0.4937	Probable ANTIGEN	30.263333	3238.647
ITRFQTLLALHRSYLLLSVGGLPFNDGVYF	0.4875	Probable ANTIGEN	20.253333	3411.945
PINLVRDLPQGFSALLLSVGGLTDEMIAQY	0.4778	Probable ANTIGEN	31.493333	3231.715
PINLVRDLPQGFSALLLSVGGLSYFIASFR	0.4777	Probable ANTIGEN	20.96	3251.773
ITRFQTLLALHRSYLLLSVGGVASQSIIAY	0.4697	Probable ANTIGEN	18.446667	3291.838
SRTLSYYKLGASQRVLLSVGGLPPAYTNSF	0.4593	Probable ANTIGEN	25.833333	3246.669
ITRFQTLLALHRSYLLLSVGGRLFRKSNLK	0.4419	Probable ANTIGEN	16.9	3502.164
SRTLSYYKLGASQRVLLSVGGLTDEMIAQY	0.4411	Probable ANTIGEN	31.81	3320.769
SRTLSYYKLGASQRVLLSVGGLSYFIASFR	0.441	Probable ANTIGEN	22.766667	3340.826
ITRFQTLLALHRSYLLLSVGGGVYFASTEK	0.4402	Probable ANTIGEN	22.63	3341.854
SYYKLGASQRVAGDSLLSVGGTISLAGSYK	0.4392	Probable ANTIGEN	23.083333	3049.39
SYYKLGASQRVAGDSLLSVGGKTFPPTEPK	0.4375	Probable ANTIGEN	14.386667	3154.527
PINLVRDLPQGFSALLLSVGGWTAGAAAYY	0.4142	Probable ANTIGEN	27.38	3121.542
SYYKLGASQRVAGDSLLSVGGLPFNDGVYF	0.4087	Probable ANTIGEN	25.596667	3181.507
ITRFQTLLALHRSYLLLSVGGLPPAYTNSF	0.4058	Probable ANTIGEN	18.183333	3349.876

Table S4. Predicted synthetic long peptide (SLPs) sequences, toxicity and allergenicity prediction. Toxicity prediction was performed using ToxinPred webserver, while allergenicity was predicted using AllerCatPro.

Peptide sequence	SVM score	Conclusion	Allergenicity
PINLVRDLPQGFSALLLSVGGAEIRASANL	-1.15	Non-Toxin	No significant hit (E-value threshold 0.001)
PINLVRDLPQGFSALLLSVGGATSRTLSYY	-1.47	Non-Toxin	No significant hit (E-value threshold 0.001)
PINLVRDLPQGFSALLLSVGGTISLAGSYK	-1.34	Non-Toxin	No significant hit (E-value threshold 0.001)
PINLVRDLPQGFSALLLSVGGKTFPPTEPK	-1.21	Non-Toxin	No significant hit (E-value threshold 0.001)
PINLVRDLPQGFSALLLSVGGTSNQVAVLY	-1.63	Non-Toxin	No significant hit (E-value threshold 0.001)
ITRFQTLLALHRSYLLLSVGGATSRTLSYY	-1.25	Non-Toxin	No significant hit (E-value threshold 0.001)
ITRFQTLLALHRSYLLLSVGGAEIRASAN	-1.03	Non-Toxin	No significant hit (E-value threshold 0.001)
SRTLSYYKLGASQRVLLSVGGTISLAGSYK	-1.25	Non-Toxin	No significant hit (E-value threshold 0.001)
SRTLSYYKLGASQRVLLSVGGKTFPPTEPK	-1.15	Non-Toxin	No significant hit (E-value threshold 0.001)
SRTLSYYKLGASQRVLLSVGGTSNQVAVLY	-1.47	Non-Toxin	No significant hit (E-value threshold 0.001)
PINLVRDLPQGFSALLLSVGGVASQSIIAY	-1.51	Non-Toxin	No significant hit (E-value threshold 0.001)
SRTLSYYKLGASQRVLLSVGGLPFNDGVYF	-1.42	Non-Toxin	No significant hit (E-value threshold 0.001)
PINLVRDLPQGFSALLLSVGGGVYFASTEK	-1.65	Non-Toxin	No significant hit (E-value threshold 0.001)
SRTLSYYKLGASQRVLLSVGGVASQSIIAY	-1.45	Non-Toxin	No significant hit (E-value threshold 0.001)
SYYKLGASQRVAGDSLLSVGGAEIRASANL	-0.99	Non-Toxin	No significant hit (E-value threshold 0.001)
ITRFQTLLALHRSYLLLSVGGTISLAGSYK	-1.15	Non-Toxin	No significant hit (E-value threshold 0.001)
ITRFQTLLALHRSYLLLSVGGKTFPPTEPK	-1.11	Non-Toxin	No significant hit (E-value threshold 0.001)
ITRFQTLLALHRSYLLLSVGGTSNQVAVLY	-1.4	Non-Toxin	No significant hit (E-value threshold 0.001)
SYYKLGASQRVAGDSLLSVGGATSRTLSYY	-1.25	Non-Toxin	No significant hit (E-value threshold 0.001)
PINLVRDLPQGFSALLLSVGGLPPAYTNSF	-1.23	Non-Toxin	No significant hit (E-value threshold 0.001)
SRTLSYYKLGASQRVLLSVGGRLFRKSNLK	-1.39	Non-Toxin	No significant hit (E-value threshold 0.001)
SRTLSYYKLGASQRVLLSVGGGVYFASTEK	-1.64	Non-Toxin	No significant hit (E-value threshold 0.001)
ITRFQTLLALHRSYLLLSVGGLPFNDGVYF	-1.4	Non-Toxin	No significant hit (E-value threshold 0.001)
PINLVRDLPQGFSALLLSVGGLTDEMIAQY	-1.1	Non-Toxin	No significant hit (E-value threshold 0.001)
PINLVRDLPQGFSALLLSVGGLSYFIASFR	-1.47	Non-Toxin	No significant hit (E-value threshold 0.001)
ITRFQTLLALHRSYLLLSVGGVASQSIIAY	-1.37	Non-Toxin	No significant hit (E-value threshold 0.001)
SRTLSYYKLGASQRVLLSVGGLPPAYTNSF	-1.2	Non-Toxin	No significant hit (E-value threshold 0.001)
ITRFQTLLALHRSYLLLSVGGRLFRKSNLK	-1.28	Non-Toxin	No significant hit (E-value threshold 0.001)

SRTLSYYKLGASQRVLLSVGGLTDEMIAQY	-1	Non-Toxin	No significant hit (E-value threshold 0.001)
SRTLSYYKLGASQRVLLSVGGLSYFIASFR	-1.4	Non-Toxin	No significant hit (E-value threshold 0.001)
ITRFQTLLALHRSYLLLSVGGGVYFASTEK	-1.49	Non-Toxin	No significant hit (E-value threshold 0.001)
SYYKLGASQRVAGDSLLSVGGTISLAGSYK	-1.02	Non-Toxin	No significant hit (E-value threshold 0.001)
SYYKLGASQRVAGDSLLSVGGKTFPPTEPK	-1.03	Non-Toxin	No significant hit (E-value threshold 0.001)
PINLVRDLPQGFSALLLSVGGWTAGAAAYY	-1.53	Non-Toxin	No significant hit (E-value threshold 0.001)
SYYKLGASQRVAGDSLLSVGGLPFNDGVYF	-1.31	Non-Toxin	No significant hit (E-value threshold 0.001)
ITRFQTLLALHRSYLLLSVGGLPPAYTNSF	-1.2	Non-Toxin	No significant hit (E-value threshold 0.001)

Table S5. Synthetic long peptide sequences and their best Rosetta score. MFR – most favourable regions; AAR – additional allowed regions.

Number	Peptide sequence	Residues in MFR (%)	Residues in AAR (%)	Rosetta score
1	PINLVRDLPQGFSALLLSVGGAEIRASANL	100	0	-66.335
2	PINLVRDLPQGFSALLLSVGGATSRTLSYY	100	0	-61.426
3	PINLVRDLPQGFSALLLSVGGTISLAGSYK	91.3	8.7	-65.283
4	PINLVRDLPQGFSALLLSVGGKTFPPTEPK	100	0	-64.665
5	PINLVRDLPQGFSALLLSVGGTSNQVAVLY	100	0	-67.906
6	ITRFQTLLALHRSYLLLSVGGATSRTLSYY	92.3	7.7	-52.653
7	ITRFQTLLALHRSYLLLSVGGAEIRASAN	96.2	3.8	-63.795
8	SRTLSYYKLGASQRVLLSVGGTISLAGSYK	95.8	4.2	-69.309
9	SRTLSYYKLGASQRVLLSVGGKTFPPTEPK	95.5	4.5	-68.514
10	SRTLSYYKLGASQRVLLSVGGTSNQVAVLY	96	4	-65.258
11	PINLVRDLPQGFSALLLSVGGVASQSIIAY	95.8	4.2	-56.234
12	SRTLSYYKLGASQRVLLSVGGLPFNDGVYF	95.7	4.3	-60.258
13	PINLVRDLPQGFSALLLSVGGGVYFASTEK	100	0	-65.994
14	SRTLSYYKLGASQRVLLSVGGVASQSIIAY	100	0	-66.636
15	SYYKLGASQRVAGDSLLSVGGAEIRASANL	95.8	4.2	-51.95
16	ITRFQTLLALHRSYLLLSVGGTISLAGSYK	96	4	-52.5
17	ITRFQTLLALHRSYLLLSVGGKTFPPTEPK	95.7	4.3	-64.15
18	ITRFQTLLALHRSYLLLSVGGTSNQVAVLY	92.3	7.7	-58.35
19	SYYKLGASQRVAGDSLLSVGGATSRTLSYY	95.8	4.2	-49.082
20	PINLVRDLPQGFSALLLSVGGLPPAYTNSF	90.9	9.1	-71.157
21	SRTLSYYKLGASQRVLLSVGGRLFRKSNLK	96	4	-62.715
22	SRTLSYYKLGASQRVLLSVGGGVYFASTEK	95.8	4.2	-65.232
23	ITRFQTLLALHRSYLLLSVGGLPFNDGVYF	95.8	4.2	-60.78
24	PINLVRDLPQGFSALLLSVGGLTDEMIAQY	95.8	4.2	-63.009
25	PINLVRDLPQGFSALLLSVGGLSYFIASFR	100	0	-58.837
26	ITRFQTLLALHRSYLLLSVGGVASQSIIAY	100	0	-54.92
27	SRTLSYYKLGASQRVLLSVGGLPPAYTNSF	95.7	4.3	-66.328
28	ITRFQTLLALHRSYLLLSVGGRLFRKSNLK	96.2	3.8	-60.846
29	SRTLSYYKLGASQRVLLSVGGLTDEMIAQY	100	0	-66.41
30	SRTLSYYKLGASQRVLLSVGGLSYFIASFR	96	4	-67.493
31	ITRFQTLLALHRSYLLLSVGGGVYFASTEK	96	4	-59.597
32	SYYKLGASQRVAGDSLLSVGGTISLAGSYK	91.3	8.7	-55.281
33	SYYKLGASQRVAGDSLLSVGGKTFPPTEPK	100	0	-53.123
34	PINLVRDLPQGFSALLLSVGGWTAGAAAYY	95.7	4.3	-65.343

35	SYYKLGASQRVAGDSLLSVGGLPFNDGVYF	95.5	4.5	-49.98
36	ITRFQTLLALHRSYLLLSVGGLPPAYTNSF	95.8	4.2	-64.887

Table S6. HADDOCK results for TLR2-SLP molecular docking (after refinement).

Complex	HADDOCK score	Van der Waals energy	Electrostatic energy	Desolvation energy	Restraints violation energy	Buried Surface Area (Å^2)
TLR2/Pep 1	-90.9 ± 0.3	-56.0 ± 2.5	-192.9 ± 16.4	3.7 ± 2.6	0.6 ± 0.2	1343.3 ± 10.2
TLR2/Pep 2	-106.4 ± 0.9	-68.1 ± 1.5	-186.4 ± 5.0	-1.1 ± 0.8	0.9 ± 0.5	1982.4 ± 33.2
TLR2/Pep 3	-85.0 ± 1.9	-51.8 ± 1.9	-200.2 ± 23.2	6.7 ± 3.6	1.6 ± 1.0	1592.2 ± 47.5
TLR2/Pep 4	-100.2 ± 1.5	-41.1 ± 4.2	-310.4 ± 13.6	3.0 ± 1.1	0.6 ± 0.2	1597.3 ± 42.7
TLR2/Pep 5	-87.9 ± 3.4	-56.8 ± 2.1	-135.7 ± 11.5	-4.0 ± 0.7	0.8 ± 0.7	1512.7 ± 42.6
TLR2/Pep 6	-125.1 ± 2.7	-72.0 ± 0.3	-210.7 ± 6.1	-11.0 ± 2.4	0.8 ± 0.5	1836.8 ± 49.8
TLR2/Pep 7	-93.9 ± 1.0	-66.0 ± 2.5	-111.3 ± 10.3	-5.7 ± 1.3	0.3 ± 0.1	1657.8 ± 15.5
TLR2/Pep 8	-116.0 ± 4.9	-58.4 ± 2.2	-289.1 ± 7.5	0.2 ± 1.7	0.8 ± 0.5	1873.1 ± 47.3
TLR2/Pep 9	-113.8 ± 2.8	-66.9 ± 2.1	-251.2 ± 2.9	3.3 ± 1.7	0.8 ± 0.2	1996.9 ± 32.2
TLR2/Pep 10	-80.4 ± 2.1	-57.8 ± 1.8	-131.3 ± 13.0	3.6 ± 3.2	1.1 ± 0.6	1527.3 ± 9.3
TLR2/Pep 11	-92.5 ± 0.8	-56.6 ± 2.4	-193.2 ± 15.1	2.6 ± 0.3	0.4 ± 0.2	1398.7 ± 12.9
TLR2/Pep 12	-122.4 ± 2.1	-69.4 ± 3.7	-272.6 ± 8.1	1.5 ± 1.5	1.0 ± 0.6	1916.3 ± 65.8
TLR2/Pep 13	-121.9 ± 1.4	-59.7 ± 3.9	-233.3 ± 16.5	-15.5 ± 1.1	0.6 ± 0.4	1921.9 ± 17.7
TLR2/Pep 14	-119.3 ± 1.2	-56.3 ± 1.6	-375.6 ± 23.6	12.0 ± 3.2	1.1 ± 0.4	1636.2 ± 37.5
TLR2/Pep 15	-107.8 ± 1.3	-65.7 ± 2.2	-173.5 ± 8.9	-7.4 ± 0.4	0.6 ± 0.2	1596.7 ± 19.6
TLR2/Pep 16	-97.1 ± 2.7	-57.4 ± 2.2	-152.4 ± 8.3	-9.3 ± 1.7	0.3 ± 0.1	1563.4 ± 66.3
TLR2/Pep 17	-100.4 ± 2.6	-59.0 ± 2.1	-231.2 ± 9.8	4.8 ± 2.4	0.6 ± 0.3	1505.1 ± 29.2
TLR2/Pep 18	-90.6 ± 1.1	-42.9 ± 2.0	-201.4 ± 13.4	-7.5 ± 3.0	0.3 ± 0.3	1288.0 ± 25.7
TLR2/Pep 19	-141.3 ± 2.8	-59.2 ± 3.0	-447.4 ± 27.2	7.3 ± 2.4	0.6 ± 0.7	1849.4 ± 23.8
TLR2/Pep 20	-73.5 ± 3.6	-55.7 ± 3.0	-93.5 ± 26.8	0.8 ± 1.5	0.4 ± 0.1	1471.0 ± 18.4
TLR2/Pep 21	-101.7 ± 1.7	-51.6 ± 4.1	-288.7 ± 26.7	7.6 ± 1.2	0.6 ± 0.2	1539.9 ± 19.4
TLR2/Pep 22	-113.3 ± 1.9	-48.5 ± 2.7	-314.0 ± 17.3	-2.2 ± 1.4	1.0 ± 0.6	1647.7 ± 14.7
TLR2/Pep 23	-130.7 ± 0.5	-65.6 ± 5.3	-216.3 ± 34.2	-22.0 ± 1.7	0.6 ± 0.1	1830.5 ± 54.5
TLR2/Pep 24	-102.4 ± 4.9	-50.8 ± 6.7	-271.9 ± 32.7	2.7 ± 1.2	0.5 ± 0.1	1478.8 ± 47.9

TLR2/Pep 25	-130.7 ± 1.6	-76.5 ± 1.0	-230.0 ± 13.3	-8.2 ± 2.5	0.6 ± 0.3	1923.1 ± 34.9
TLR2/Pep 26	-103.4 ± 1.8	-62.3 ± 4.4	-152.1 ± 4.0	-10.8 ± 2.1	0.5 ± 0.1	1853.0 ± 78.1
TLR2/Pep 27	-110.2 ± 2.8	-65.1 ± 4.7	-181.6 ± 11.8	-8.9 ± 1.6	1.1 ± 0.8	1623.3 ± 24.9
TLR2/Pep 28	-119.6 ± 2.7	-47.7 ± 3.2	-341.8 ± 11.4	-3.7 ± 1.7	0.5 ± 0.4	1584.6 ± 39.4
TLR2/Pep 29	-91.3 ± 1.3	-49.3 ± 2.2	-221.1 ± 10.3	2.1 ± 1.3	0.4 ± 0.3	1580.4 ± 26.6
TLR2/Pep 30	-126.2 ± 0.8	-69.0 ± 2.6	-258.1 ± 13.9	-5.6 ± 0.6	0.6 ± 0.6	1915.4 ± 34.8
TLR2/Pep 31	-135.5 ± 3.0	-63.4 ± 2.7	-317.5 ± 22.6	-8.6 ± 1.9	0.4 ± 0.2	1767.9 ± 32.0
TLR2/Pep 32	-97.9 ± 2.0	-39.3 ± 4.4	-284.3 ± 28.9	-1.8 ± 1.1	0.5 ± 0.2	1235.6 ± 12.3
TLR2/Pep 33	-110.5 ± 3.1	-62.2 ± 4.1	-239.6 ± 17.7	-0.5 ± 1.7	0.7 ± 0.2	1769.4 ± 22.4
TLR2/Pep 34	-94.0 ± 2.4	-63.5 ± 3.2	-117.9 ± 13.6	-7.0 ± 1.7	0.7 ± 0.4	1599.6 ± 52.5
TLR2/Pep 35	-137.3 ± 3.8	-51.9 ± 3.5	-471.3 ± 9.2	8.8 ± 2.3	0.8 ± 0.4	1989.9 ± 60.4
TLR2/Pep 36	-98.2 ± 4.3	-58.6 ± 4.0	-130.8 ± 12.6	-13.5 ± 2.0	0.7 ± 0.5	1495.2 ± 36.6

Table S7. HADDOCK results for TLR4-SLP molecular docking (after refinement).

Complex	HADDOCK score	Van der Waals energy	Electrostatic energy	Desolvation energy	Restraints violation energy	Buried Surface Area (Å^2)
TLR4/Pep1	-87.7 ± 1.2	-48.2 ± 1.8	-95.1 ± 12.5	-20.7 ± 0.6	1.8 ± 0.9	1250.5 ± 7.4
TLR4/Pep2	-109.9 ± 1.1	-54.4 ± 2.4	-154.5 ± 11.4	-24.8 ± 1.1	1.2 ± 0.4	1299.9 ± 30.8
TLR4/Pep3	-97.0 ± 0.8	-60.2 ± 1.5	-67.1 ± 12.1	-23.6 ± 1.3	2.4 ± 0.9	1349.3 ± 12.0
TLR4/Pep4	-90.8 ± 0.7	-48.9 ± 1.8	-77.1 ± 9.8	-26.6 ± 1.0	1.0 ± 0.5	1135.0 ± 32.3
TLR4/Pep5	-97.3 ± 0.7	-57.2 ± 2.6	-110.9 ± 7.8	-18.0 ± 1.8	1.3 ± 0.2	1407.2 ± 25.3
TLR4/Pep6	-122.7 ± 1.0	-64.4 ± 1.6	-152.7 ± 13.8	-27.9 ± 1.3	1.6 ± 0.4	1422.5 ± 35.7
TLR4/Pep7	-113.5 ± 0.5	-52.7 ± 1.6	-135.6 ± 9.8	-33.7 ± 1.1	0.9 ± 0.4	1345.7 ± 12.6
TLR4/Pep8	-102.8 ± 2.4	-55.4 ± 2.9	-137.6 ± 12.6	-20.0 ± 1.6	1.3 ± 0.6	1229.5 ± 17.7
TLR4/Pep9	-108.9 ± 3.7	-56.3 ± 3.5	-170.6 ± 23.3	-18.6 ± 1.7	1.5 ± 0.5	1378.0 ± 20.9
TLR4/Pep10	-98.1 ± 5.9	-52.7 ± 3.9	-103.1 ± 19.4	-25.0 ± 1.1	1.8 ± 0.5	1361.8 ± 35.5
TLR4/Pep11	-80.8 ± 0.8	-48.9 ± 1.7	-82.6 ± 14.1	-15.5 ± 1.1	1.2 ± 0.3	1195.2 ± 37.6
TLR4/Pep12	-128.7 ± 1.3	-59.8 ± 3.9	-186.9 ± 16.5	-31.7 ± 2.1	1.4 ± 0.3	1421.4 ± 32.2
TLR4/Pep13	-112.2 ± 1.4	-57.5 ± 4.4	-124.1 ± 14.8	-30.1 ± 1.5	2.0 ± 0.2	1548.8 ± 35.0
TLR4/Pep14	-109.9 ± 0.7	-56.2 ± 0.6	-173.3 ± 8.7	-19.1 ± 1.0	1.1 ± 0.4	1325.4 ± 27.0
TLR4/Pep15	-119.9 ± 5.0	-55.4 ± 4.5	-257.3 ± 41.8	-13.2 ± 2.1	1.8 ± 0.2	1409.5 ± 15.0
TLR4/Pep16	-109.6 ± 1.6	-49.0 ± 1.3	-192.8 ± 10.8	-22.1 ± 1.0	1.2 ± 0.6	1465.0 ± 23.0
TLR4/Pep17	-125.2 ± 1.6	-51.9 ± 2.2	-224.9 ± 14.0	-28.5 ± 1.8	1.5 ± 0.3	1304.1 ± 34.2
TLR4/Pep18	-100.9 ± 0.7	-52.9 ± 0.3	-92.0 ± 2.8	-29.7 ± 0.5	1.0 ± 0.2	1321.4 ± 26.2
TLR4/Pep19	-129.0 ± 1.5	-73.1 ± 2.2	-152.2 ± 9.9	-25.6 ± 3.2	1.2 ± 0.6	1780.1 ± 43.9
TLR4/Pep20	-84.0 ± 2.0	-51.8 ± 1.6	-56.8 ± 5.2	-20.9 ± 1.0	0.8 ± 0.2	1129.6 ± 36.7
TLR4/Pep21	-105.4 ± 2.8	-54.5 ± 1.0	-152.8 ± 11.2	-20.5 ± 2.2	2.2 ± 0.9	1347.1 ± 25.0
TLR4/Pep22	-108.2 ± 5.2	-50.3 ± 1.2	-164.4 ± 23.9	-25.1 ± 0.9	0.8 ± 0.3	1199.0 ± 34.2

TLR4/Pep23	-121.8 ± 0.6	-52.9 ± 1.4	-155.8 ± 6.3	-37.8 ± 0.2	1.4 ± 0.3	1371.0 ± 8.7
TLR4/Pep24	-95.6 ± 0.5	-47.3 ± 2.0	-145.8 ± 12.2	-19.2 ± 1.6	1.4 ± 0.6	1169.3 ± 11.8
TLR4/Pep25	-116.9 ± 1.8	-67.8 ± 1.8	-85.3 ± 6.5	-32.1 ± 1.6	1.1 ± 0.4	1421.9 ± 24.0
TLR4/Pep26	-115.6 ± 2.1	-64.0 ± 2.7	-39.0 ± 2.2	-43.9 ± 0.8	1.5 ± 0.4	1317.9 ± 44.4
TLR4/Pep27	-107.0 ± 1.6	-48.9 ± 0.8	-159.3 ± 11.3	-26.3 ± 2.2	1.1 ± 0.4	1271.2 ± 22.1
TLR4/Pep28	-116.1 ± 0.4	-59.2 ± 1.1	-135.0 ± 10.8	-29.9 ± 1.9	0.8 ± 0.3	1390.9 ± 19.4
TLR4/Pep29	-106.5 ± 1.6	-57.4 ± 2.6	-122.0 ± 9.4	-24.8 ± 1.6	1.4 ± 0.6	1293.1 ± 20.9
TLR4/Pep30	-113.9 ± 0.5	-58.1 ± 1.3	-73.2 ± 11.5	-41.2 ± 1.2	0.9 ± 0.3	1301.3 ± 21.1
TLR4/Pep31	-102.9 ± 0.7	-42.2 ± 1.9	-158.9 ± 9.2	-29.1 ± 1.8	1.4 ± 0.7	1155.5 ± 15.1
TLR4/Pep32	-122.0 ± 1.1	-65.9 ± 0.8	-186.0 ± 10.0	-18.9 ± 0.7	0.9 ± 0.3	1288.1 ± 13.5
TLR4/Pep33	-120.8 ± 3.1	-61.0 ± 5.2	-140.4 ± 16.3	-31.8 ± 1.3	1.4 ± 0.4	1368.2 ± 43.1
TLR4/Pep34	-130.8 ± 4.5	-69.5 ± 2.6	-108.3 ± 7.7	-39.7 ± 1.6	1.2 ± 0.5	1587.9 ± 38.8
TLR4/Pep35	-144.2 ± 1.6	-68.6 ± 2.2	-242.2 ± 17.9	-27.4 ± 1.9	1.7 ± 0.7	1659.2 ± 22.7
TLR4/Pep36	-105.7 ± 1.8	-55.7 ± 1.0	-153.7 ± 14.5	-19.4 ± 0.7	1.5 ± 0.5	1369.9 ± 32.4

Table S8. Predicted ΔG and Kd for the TLR2/4-SLP interactions using PRODIGY.

Complex	ΔG(kcal/mol)	K_d(M) at 37.0 ^{o}C	Complex	ΔG(kcal/mol)	K_d(M) at 37.0 ^{o}C
TLR2/Pep1	-9.7	1.5×10^{-7}	TLR4/Pep1	-9	4.9×10^{-7}
TLR2/Pep2	-12.5	1.6×10^{-9}	TLR4/Pep2	-10.4	4.6×10^{-8}
TLR2/Pep3	-11.8	4.6×10^{-9}	TLR4/Pep3	-11.3	1.0×10^{-8}
TLR2/Pep4	-10.8	2.3×10^{-8}	TLR4/Pep4	-8.9	5.5×10^{-7}
TLR2/Pep5	-12	3.5×10^{-9}	TLR4/Pep5	-11.4	9.1×10^{-9}
TLR2/Pep6	-10.9	1.9×10^{-8}	TLR4/Pep6	-10.1	7.3×10^{-8}
TLR2/Pep7	-10.7	2.6×10^{-8}	TLR4/Pep7	-9.6	1.7×10^{-7}
TLR2/Pep8	-11.5	8.1×10^{-9}	TLR4/Pep8	-10.8	2.5×10^{-8}
TLR2/Pep9	-11.7	5.7×10^{-9}	TLR4/Pep9	-9.7	$1.4 \times 10_{-7}$
TLR2/Pep10	-11.2	1.4×10^{-8}	TLR4/Pep10	-9.5	2.1×10^{-7}
TLR2/Pep11	-10	8.3×10^{-8}	TLR4/Pep11	-10.8	2.6×10^{-8}
TLR2/Pep12	-13	7.1×10^{-10}	TLR4/Pep12	-9.8	1.3×10^{-7}
TLR2/Pep13	-12.2	2.5×10^{-9}	TLR4/Pep13	-11.5	7.9×10^{-9}
TLR2/Pep14	-11.9	4.0×10^{-9}	TLR4/Pep14	-10.2	6.7×10^{-8}
TLR2/Pep15	-12	3.5×10^{-9}	TLR4/Pep15	-10.8	2.5×10^{-8}
TLR2/Pep16	-11.6	7.1×10^{-9}	TLR4/Pep16	-9.7	1.4×10^{-7}
TLR2/Pep17	-10.9	2.2×10^{-8}	TLR4/Pep17	-9.5	2.1×10^{-7}
TLR2/Pep18	-9.9	1.1×10^{-7}	TLR4/Pep18	-10.8	2.4×10^{-8}
TLR2/Pep19	-11.6	6.9×10^{-9}	TLR4/Pep19	-12.3	2.2×10^{-9}
TLR2/Pep20	-11.2	1.3×10^{-8}	TLR4/Pep20	-9.2	3.1×10^{-7}
TLR2/Pep21	-10.8	2.4×10^{-8}	TLR4/Pep21	-10.5	3.7×10^{-8}
TLR2/Pep22	-11.1	1.4×10^{-8}	TLR4/Pep22	-10.6	3.4×10^{-8}
TLR2/Pep23	-11.8	4.5×10^{-9}	TLR4/Pep23	-9.8	1.1×10^{-7}
TLR2/Pep24	-10.4	4.7×10^{-8}	TLR4/Pep24	-9.4	2.2×10^{-7}
TLR2/Pep25	-12.8	9.1×10^{-10}	TLR4/Pep25	-12.8	9.1×10^{-10}
TLR2/Pep26	-12.3	2.2×10^{-9}	TLR4/Pep26	-9.3	2.7×10^{-7}
TLR2/Pep27	-13.3	4.5×10^{-10}	TLR4/Pep27	-10	8.6×10^{-8}
TLR2/Pep28	-11.5	7.7×10^{-9}	TLR4/Pep28	-9.7	1.4×10^{-7}
TLR2/Pep29	-11.4	9.0×10^{-9}	TLR4/Pep29	-10.7	2.9×10^{-8}
TLR2/Pep30	-13.1	6.1×10^{-10}	TLR4/Pep30	-10.6	3.2×10^{-8}
TLR2/Pep31	-11.2	1.3×10^{-8}	TLR4/Pep31	-9.2	3.2×10^{-7}
TLR2/Pep32	-10.1	8.0×10^{-8}	TLR4/Pep32	-9.7	1.4×10^{-7}
TLR2/Pep33	-10.6	3.1×10^{-8}	TLR4/Pep33	-10.1	8.0×10^{-8}

TLR2/Pep34	-12.1	2.9×10^{-9}	TLR4/Pep34	-12.5	1.5×10^{-9}
TLR2/Pep35	-12.5	1.6×10^{-9}	TLR4/Pep35	-10.5	3.7×10^{-8}
TLR2/Pep36	-11	1.7×10^{-8}	TLR4/Pep36	-10.7	3.1×10^{-8}
